# Oxidation of independent and combined ingested galactose and glucose during exercise

**DOI:** 10.1152/japplphysiol.00105.2022

**Published:** 2022-10-06

**Authors:** Oliver J. Odell, Samuel G. Impey, Brandon J. Shad, Tim Podlogar, Rafael B. Salgueiro, David S. Rowlands, Gareth A. Wallis

**Affiliations:** ^1^School of Sport, Exercise and Rehabilitation Sciences, College of Life and Environmental Sciences, University of Birmingham, Birmingham, United Kingdom; ^2^Department of Physiology and Biophysics, University of Sao Paulo, Sao Paulo, Brazil; ^3^School of Sport, Exercise and Nutrition, Massey University, Auckland, New Zealand

**Keywords:** metabolism, nutrition, physical activity, substrate oxidation, sugars

## Abstract

Coingestion of glucose and galactose has been shown to enhance splanchnic extraction and metabolism of ingested galactose at rest; effects during exercise are unknown. This study examined whether combined ingestion of galactose and glucose during exercise enhances exogenous galactose oxidation. Fourteen endurance-trained male and female participants [age, 27 (5) yr; V̇o_2peak_, 58.1 (7.0) mL·kg^−1^·min^−1^] performed cycle ergometry for 150 min at 50% peak power on four occasions, in a randomized counterbalanced manner. During exercise, they ingested beverages providing carbohydrates at rates of 0.4 g.min^−1^ galactose (GAL), 0.8 g.min^−1^ glucose (GLU), and on two occasions 0.8 g.min^−1^ total galactose-glucose (GAL + GLU; 1:1 ratio). Single-monosaccharide ^13^C-labeling (*) was used to calculate independent (GAL, GLU, GAL* + GLU, and GAL + GLU*) and combined (GAL* + GLU*, COMBINE) exogenous-monosaccharide oxidation between exercise. Plasma galactose concentrations with GAL + GLU [0.4 mmol.L; 95% confidence limits (CL): 0.1, 0.6] were lower (contrast: 0.5 mmol.L; 95% CL: 0.2, 0.8; *P* < 0.0001) than when GAL alone (0.9 mmol.L; 95% CL: 0.7, 1.2) was ingested. Exogenous carbohydrate oxidation with GAL alone (0.31 g·min^−1^; 95% CL: 0.28, 0.35) was marginally reduced (contrast: 0.05 g·min^−1^; 95% CL: −0.09, 0.00007; *P* = 0.01) when combined with glucose (GAL* + GLU 0.27 g·min^−1^; 0.24, 0.30). Total combined exogenous-carbohydrate oxidation (COMBINE: 0.57 g·min^−1^; 95% CL: 0.49, 0.64) was similar (contrast: 0.02 g·min^−1^; 95% CL: −0.05, 0.09; *P* = 0.63) when compared with isoenergetic GLU (0.55 g·min^−1^; 95% CL: 0.52, 0.58). In conclusion, coingestion of glucose and galactose did not enhance exogenous galactose oxidation during exercise. When combined, isoenergetic galactose-glucose ingestion elicited similar exogenous-carbohydrate oxidation to glucose suggesting galactose-glucose blends are a valid alternative for glucose as an exogenous-carbohydrate source during exercise.

**NEW & NOTEWORTHY** Glucose and galactose coingestion blunted the galactosemia seen with galactose-only ingestion during exercise. Glucose and galactose coingestion did not enhance the oxidation of ingested galactose during exercise. Combined galactose-glucose (1:1 ratio) ingestion was oxidized to a similar extent as isoenergetic glucose-only ingestion during exercise. Galactose-glucose blends are a viable exogenous carbohydrate energy source for ingestion during exercise.

## Graphical abstract

**Figure:**
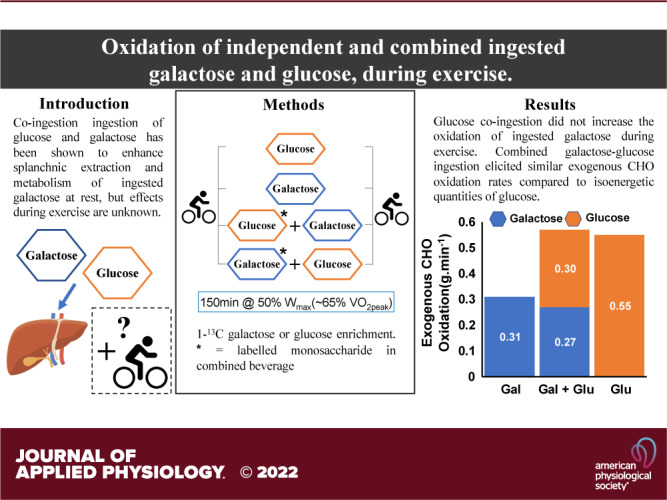

## INTRODUCTION

Carbohydrate feeding during exercise is a well-established ergogenic aid for endurance sport providing an exogenous fuel that maintains carbohydrate oxidation rates and euglycemia, can lower endogenous glycogen utilization, and positively influences central nervous system function (1, 2). Exogenous carbohydrate oxidation (CHO_exo_) is commonly measured to determine the ergogenic potential of ingested carbohydrates and is known to be mostly influenced by carbohydrate dose (3) and type (4, 5). CHO_exo_ with glucose (or glucose polymer) feeding increases in a dose-dependent manner and plateaus at peak oxidation rates ranging from 0.5 to 1.1 g·min^−1^ when ingested at rates ≥1.0–1.2 g·min^−1^ (6, 7). The rate-limiting step in exogenous glucose oxidation is believed to be intestinal epithelial absorption via the sodium-glucose-linked transporter (SGLT1) (5, 8). Of the two other monosaccharides, fructose has been observed to be oxidized at up to 25% lower rates than glucose (7, 9, 10), whereas the oxidation of galactose is as low as 50%–60% of glucose. This is despite galactose being absorbed using the same intestinal epithelial transporters as glucose (9, 11). Therefore, galactose has been considered an inferior exogenous fuel for exercise (7).

The slower relative oxidation of ingested galactose during exercise has been attributed to reduced intestinal absorption, slow hepatic gluconeogenesis from galactose via the Leloir pathway, or storage as liver glycogen rather than oxidation (9, 11). The Leloir pathway is thought to be the primary route for galactose clearance and results in the production of glucose-1-phosphate in the liver. Previously, marked increases in plasma galactose concentrations (i.e., galactosemia) were observed when healthy men ingested exclusively galactose at rates of ∼1.2 g·min^−1^ during cycling exercise, which suggests a limitation in liver galactose clearance capacity, rather than intestinal absorption (11, 12). Galactosemia observed with galactose ingestion at rest is lowered with glucose coingestion, either as free glucose or as glucose contained within lactose (13). The attenuated galactosemia at rest has been proposed to be due to enhanced splanchnic galactose extraction, which following conversion to glucose may be stored as hepatic glycogen (14). During exercise, we recently showed that lactose ingested at 0.8 g·min^−1^ was oxidized at a rate comparable with the readily oxidizable carbohydrate sucrose (15), suggesting that the presence of galactose was not limiting to CHO_exo_. Based on the potential for glucose to enhance splanchnic galactose extraction and galactose-to-glucose conversion at rest and the previously observed metabolic availability of lactose during exercise, it is not unreasonable to suggest that glucose and galactose coingestion during exercise could augment the oxidation of ingested galactose.

Therefore, the objective of the present study was to test the hypothesis that combined glucose and galactose coingestion would increase CHO_exo_ from ingested galactose during exercise. A further aim was to compare the effects of combined galactose-glucose ingestion with isoenergetic quantities of glucose on CHO_exo_ during exercise.

## METHODS

### Experimental Design

Participants completed five visits to the laboratory, including a screening visit and four experimental trials. Experimental trials were separated by at least 5 days [means (SD): 11 (7) days; maximum 33 days] and were performed in a randomized order using a Latin Square design. Female participants were eligible to participate if they were normally and regularly menstruating or using hormonal contraceptives but were ineligible if they self-reported irregular or absence of menstrual cycles. Those that enrolled in the study self-reported normal menstrual cycles or use of monophasic hormonal contraception. These participants completed experimental trials during the self-reported midfollicular phase of the menstrual cycle, or during the active pill consumption phase if using monophasic hormonal contraception. Experimental trials involved 150 min of steady-state exercise on a cycle ergometer at an intensity equivalent to 50% peak power (W_max_). During exercise, participants ingested one of four carbohydrate beverages, enriched with ^13^C tracers to permit measurement of CHO_exo_, in a single-blind manner. The beverages included galactose (GAL), glucose (GLU), and two trials with galactose + glucose (GAL* + GLU and GAL + GLU*; where * indicates the ^13^C-labeled carbohydrate with the combined galactose-glucose beverages). Indirect calorimetry measurements were taken with expired breath and venous blood samples collected throughout the exercise to characterize substrate oxidation and metabolic responses to the nutritional interventions.

### Participant Characteristics

Sixteen participants, all of whom participated regularly in endurance-type activity, were recruited to the study. Fourteen (11 males, 3 females) of whom completed all experimental trials, and two participants withdrew from the study due to scheduling constraints and the time commitment required. Participants were classified as healthy by completion of a general health questionnaire, which confirmed no history of cardiometabolic disease, galactosemia, or relevant food intolerances. Additional inclusion criteria included completing endurance-type exercise of at least 30 min ≥3 times per week, with one exercise bout of ≥90 min in the previous 6 wk; and a V̇o_2peak_ of ≥50 mL^−1^·kg^−1^·min^−1^ or ≥55 mL·kg^−1^·min^−1^ for female and male participants, respectively. Participant characteristics are provided in Table 1, with participants on average being categorized at performance level 3 (i.e., trained) or above, according to established criteria (16, 17). Participants gave their written informed consent to participate in the study, which was approved by the Science, Technology, Engineering and Mathematics Ethics Committee, University of Birmingham, Birmingham, United Kingdom.

**Table 1.** Participant characteristics

Variable	Males	Females	Overall
Age, yr	27 (5)	25 (6)	27 (5)
Height, cm	180.5 (6.2)	173.8 (2.8)	179.1 (6.2)
Body mass, kg	72.1 (8.5)	62.2 (3.8)	70.6 (8.7)
V̇o_2peak_, mL·kg^−1^·min^−1^	59.9 (6.8)	51.5 (2.3)	58.1 (7.0)
Peak power, W·kg^−1^	5.1 (0.8)	5.0 (1.0)	5.1 (0.8)

Data are represented as means (SD). Males, *n* = 11; females, *n* = 3; overall, *n* = 14.

### Screening Visit

Participants attended a screening visit and gave their written informed consent before completing a general health questionnaire. Participants’ height (Stadiometer Model 220, Seca, Germany) and body mass (Champ II, OHAUSE, Switzerland) were recorded before exercise. Participants mounted a cycle ergometer (Lode Excalibur Sport, Groningen, The Netherlands) and adjusted the saddle and handlebar positioning until comfortable, which was replicated for subsequent visits. They then performed a step-incremental exercise test to exhaustion, which commenced at 100 W and was increased by 30 W every 2 min until volitional exhaustion, or until a cadence of >50 rpm could not be maintained. Indirect calorimetry was performed throughout exercise using an online automated gas analyzer (Vyntus, Vyaire Medical, Mettawa, IL), to determine V̇o_2_ and V̇co_2_. The volume transducer, CO_2,_ and O_2_ sensors were calibrated before each measurement as per the manufacturer’s instructions. Heart rate (HR) was measured continuously via telemetry (Polar H7, Kempele, Finland). Peak power was calculated as the power output of the last completed stage, added to the fraction of the time spent in the following stage, multiplied by 30 W. V̇o_2peak_ was calculated as the highest 30 s average of V̇o_2_.

#### Preexperimental control.

Participants were provided with a list of food and drinks with high natural abundances of ^13^C and were asked to avoid their consumption for the 5 days preceding experimental trials to minimize the background shift in breath ^13^CO_2_ during exercise. Participants also refrained from caffeine and alcohol for 24 h before visits. Participants recorded all meals and snacks in the 24-h before experimental trials and replicated this diet before subsequent visits.

#### Experimental trials.

Participants arrived at the laboratory in an overnight fasted state between 0700 and 0800 h and on arrival were asked to visit the toilet and void. An intravenous cannula (Venflon, Becton- Dickinson, Helsingborg, Sweden) was inserted into an antecubital vein and was attached to a three-way stopcock (Connecta, Becton Dickinson, Helsingborg, Sweden) to allow for blood sampling (10 mL) at rest, and for repeated blood sampling every 30 min during exercise. Blood was dispensed into EDTA-containing vacutainers before centrifugation at 4°C and 1,000 *g* for 15 min and storage at –70°C. Participants mounted the cycle ergometer and a resting breath sample was collected into 10-mL Exetainer tubes (Labco, High Wycombe, UK), which were filled from a mixing chamber to determine the expired breath ^13^C enrichment at rest and every 15 min during exercise. Participants then commenced 150 min of cycling at 50% W_max_ [172 (18) W] (actual mean intensities achieved were ∼65% V̇o_2peak_). Breath-by-breath respiratory gas was collected during exercise in the final 3 min of each 15 min sample period to determine V̇o_2_ and V̇co_2_. In addition, every 15 min, heart rate (HR), the rate of perceived exertion (RPE) (19), and perception of gastrointestinal (GI) discomfort on a 10-point Likert scale, described as nausea, stomach fullness, and cramping were measured (15).

### Carbohydrate Beverages

During exercise, participants consumed one of four 5% carbohydrate beverages. A 5% solution was used to be consistent with previous work (20) and carbohydrate concentrations of 5%–8% result in lower GI symptoms compared with higher concentrations (21). A 600-mL bolus of the beverage was provided at exercise onset, followed by 200-mL doses of the test beverage every 15 min thereafter, so that the total fluid intake was 2.4 L. Beverages delivered total carbohydrate at an average rate of 24 g·h^−1^ (0.4 g·min^−1^) in the GAL condition (Galaxtra, Solace Nutrition, CT), and 48 g·h^−1^ (0.8 g·min^−1^) in the GLU (Roquette, Lestrem, France), GAL*+GLU and GAL + GLU* conditions, with the latter two providing glucose and galactose in a 1:1 ratio. The quantities of carbohydrate ingested in the GLU, GAL* + GLU, and GAL + GLU* conditions are congruent with guidelines for carbohydrate ingestion during exercise of this duration (2), but such that limitations in gastrointestinal transport would not be expected to confound the experimental outcomes.

Stable (^13^C) isotope techniques were used to quantify CHO_exo_. The natural ^13^C abundance of the galactose and glucose powders used in the beverages was −23.2% and −27.2 ‰ versus Pee Dee Bellemnitella (PDB), respectively. Beverages were further enriched by the addition of 1-^13^c-galactose (99%, Cambridge Isotope Laboratories Inc., MA) in GAL and GAL* + GLU. 1-^13^c-glucose (98%–99%, Cambridge Isotopes) was added in GLU and GAL + GLU*. The final enrichment of the GAL drink was 64.9 δ‰ versus PDB and the enrichment of the galactose component of the GAL* + GLU drink was 137.8 δ‰ versus PDB (^12^C:^13^C ratio analysis by elemental analyzer IRMS, 20-20, Europa Scientific, Crewe, UK). The final enrichment of the GLU drink was 116.7 δ‰ versus PDB and the enrichment of the glucose component of the GAL + GLU* drink was 125.0 δ‰ versus PDB. Selective isotope labeling of galactose in GAL* + GLU permitted direct assessment of the effect of adding glucose on galactose oxidation through comparison of CHO_exo_ in GAL*+GLU with GAL. Selective isotope labeling of glucose in GAL + GLU* allowed calculation of exogenous-glucose oxidation in the presence of galactose. The sum of independently determined exogenous galactose and glucose oxidation from GAL* + GLU and GAL + GLU*, respectively, corresponds to the cumulative CHO_exo_ (COMBINE) from the combined galactose-glucose beverages. This selective labeling approach has been used previously to determine the oxidation rates of different carbohydrates in the same beverage during exercise (22).

#### Plasma and breath analyses.

Plasma was analyzed for glucose, lactate, nonesterified fatty acids (NEFA), and glycerol with commercially available kits (Glucose kit, Lactate kit, NEFA kit, Glycerol kit; Randox, London, UK) using an automated photometric clinical chemistry analyzer RX Daytona+ (Randox, London, UK). Plasma galactose concentration was determined using a colorimetric assay (Galactose Assay Kit, Sigma Aldrich, St Louis. MO) and insulin using an enzyme-linked immunosorbent assay (Ultrasensitive Insulin, Mercodia, Uppsala Sweden). Breath samples were analyzed for ^13^C:^12^C ratio by gas chromatography isotope ratio mass spectrometry (IRMS), Hydra 20-20, Europa Scientific, Crewe, UK).

### Calculations

Total fat and carbohydrate oxidation (CHO_tot_) were calculated using previously described equations (22). Mean substrate utilization rates were calculated using the 60–150 min time period, when recovery of ^13^CO_2_ is derived exclusively from the oxidation of ingested carbohydrates, and dilution in the bicarbonate pool becomes negligible (23). Isotopic enrichment of breath and beverage samples was expressed as δ per milliliter difference between the sample ^13^C:^12^C ratio and a laboratory reference standard, using a standard formula (20, 24). ^δ^C was then related to the international standard PDB. CHO_exo_ in GAL and GLU was determined using the following formula (22):

$${\text{CHO}}_{\text{exo}}={\;\dot{\text{V}}\text{CO}}_{2}\;[\frac{\delta^{\text{Exp}}-{\text{ δExp}}_{\text{bkg}}}{\delta^{\text{Ing}}-{\text{ δExp}}_{\text{bkg}}}]/k,$$

where δ^Exp^ is the ^13^C enrichment of the expired air, δExp_bkg_ is the median breath ^13^C enrichment in the corresponding background trial at the corresponding time-point, δ^Ing^ is the ^13^C enrichment of the ingested beverage and *k* is the amount of CO_2_ produced by the complete oxidation of 1 g of glucose (0.7426 L). The corresponding background trial included a subset of participants (*n* = 4) who completed equivalent GAL, GLU, and GAL + GLU trials (involving expired breath sampling only) consuming galactose and/or glucose without additional tracer enrichment. The average breath ^13^C for each condition was applied to all participants to further minimize the influence of background shifts on the estimation of CHO_exo_ (20). Endogenous carbohydrate oxidation (CHO_endo_) was calculated by subtracting CHO_exo_ from CHO_tot_.

CHO_exo_ of the galactose or glucose component in GAL* + GLU or GAL + GLU*, respectively, was determined as follows (22):

$$\text{Exogenous galactose oxidation}={\;\dot{\text{V}}\text{CO}}_{2}\;[\frac{{\text{δExp}}_{{\text{GAL}}^{\text{*}}\text{+GLU}}-{\text{ δExp}}_{\text{bkg}}}{{\text{δIng}}_{{\text{GAL}}^{\text{*}}}-{\text{ δIng}}_{\text{gal}}}]/k,$$

$$\text{Exogenous glucose oxidation}={\;\dot{\text{V}}\text{CO}}_{2}\;[\frac{{\text{δExp}}_{{\text{GAL+GLU}}^{\text{*}}}-{\text{ δExp}}_{\text{bkg}}}{{\text{δIng}}_{{\text{GLU}}^{\text{*}}}-{\text{ δIng}}_{\text{glu}}}]/k,$$

where δ^Exp^ is the ^13^C enrichment of the expired air, δExp_bkg_ is the average breath ^13^C enrichment in the corresponding background trial at the corresponding time point, δIng_GAL*_ and δIng_GLU*_ are the ^13^C enrichments of the labeled monosaccharides in the ingested beverage and δIng_gal_ and δIng_glu_ are the ^13^C enrichments of the unlabeled monosaccharide in the ingested beverage.

### Statistics

A sample size estimation was made with average CHO_exo_ rate as the primary outcome. Data from this laboratory showed that during comparable exercise, two trials (*n* = 8) with glucose-based carbohydrate ingestion at 108 g·h^−1^ resulted in a mean exogenous glucose oxidation rate of 0.86 (0.25) and 0.87 (0.31) g·min^−1^ for each trial, and a typical error of 0.12 g·min^−1^. Similarly, exogenous galactose oxidation was shown to have a coefficient of variation of 31% (9). The primary objective was to test if the addition of glucose to galactose enhanced the oxidation of galactose. Therefore, using equations for crossover trials (25, 26) controlling for type 1 error (α = 0.05) at 5%, and type 2 error of 20% (β = 0.8), a sample size of 16 (4 × 4 Latin square crossover) provided power to detect the smallest critical value of change in exogenous galactose oxidation of 0.091 g·min^−1^ [95% confidence limits (CL), 0.0, 0.018], which represented a 10% increase in rate of galactose oxidation in GAL + GLU versus GAL, or a small standardized effect of 0.34 × SD.

The effects of treatment on outcomes were estimated from repeated-measures linear mixed model analysis of variance (Proc Mixed, SAS 9.4, Cary, NC). Fixed effects were treatment, period, sample time point, and sex; subject was included as a random effect, with additional random variation specified within the model for period = 1 and sex = female. CHO_exo_ for COMBINE was derived within the model (retaining the native sample variance structure) from summation of the linear mixed model estimates for exogenous galactose oxidation and exogenous glucose oxidation in GAL*+GLU and GAL + GLU*, respectively. CHO_endo_ in COMBINE was derived by subtracting the independent variable raw exogenous galactose oxidation and exogenous glucose oxidation in GAL*+GLU GAL + GLU*, respectively, from the average CHO_tot_ derived from the linear mixed model for those two treatments. Estimates were followed with adjustment for multiplicity using the step-down Holm-simulated procedure for adjustment of *P* values and 95% CL (27), with statistical significance accepted at *P* < 0.05 with respect to the adjusted *P* value. Substrate utilization and blood metabolite data are presented within figures as raw means (SD) for the 60–150 min and 30–150 min periods, respectively. Statistical summaries are presented in tabular form expressed as least squares means, difference estimates, and 95% CL.

## RESULTS

### Exogenous and Endogenous Substrate Utilization

**Figure 1.**
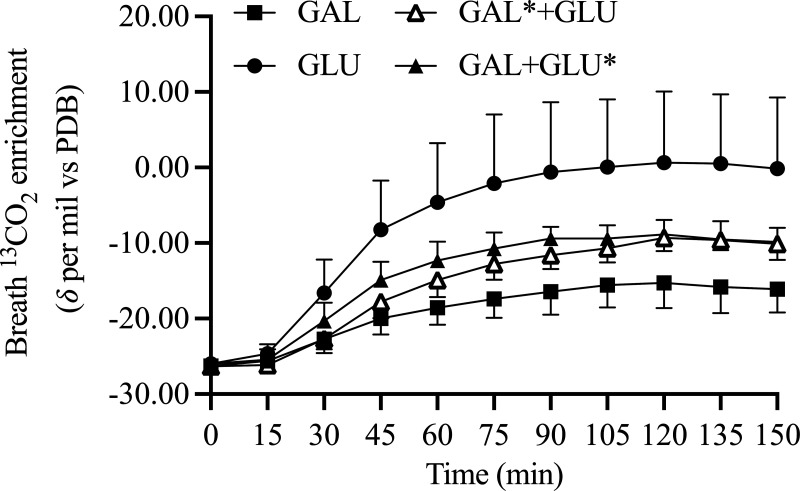
Breath ^13^CO_2_ enrichment during 150 min exercise at 50%W_max_. *n* = 14 (11 male participants, 3 female participants).

**Figure 2.**
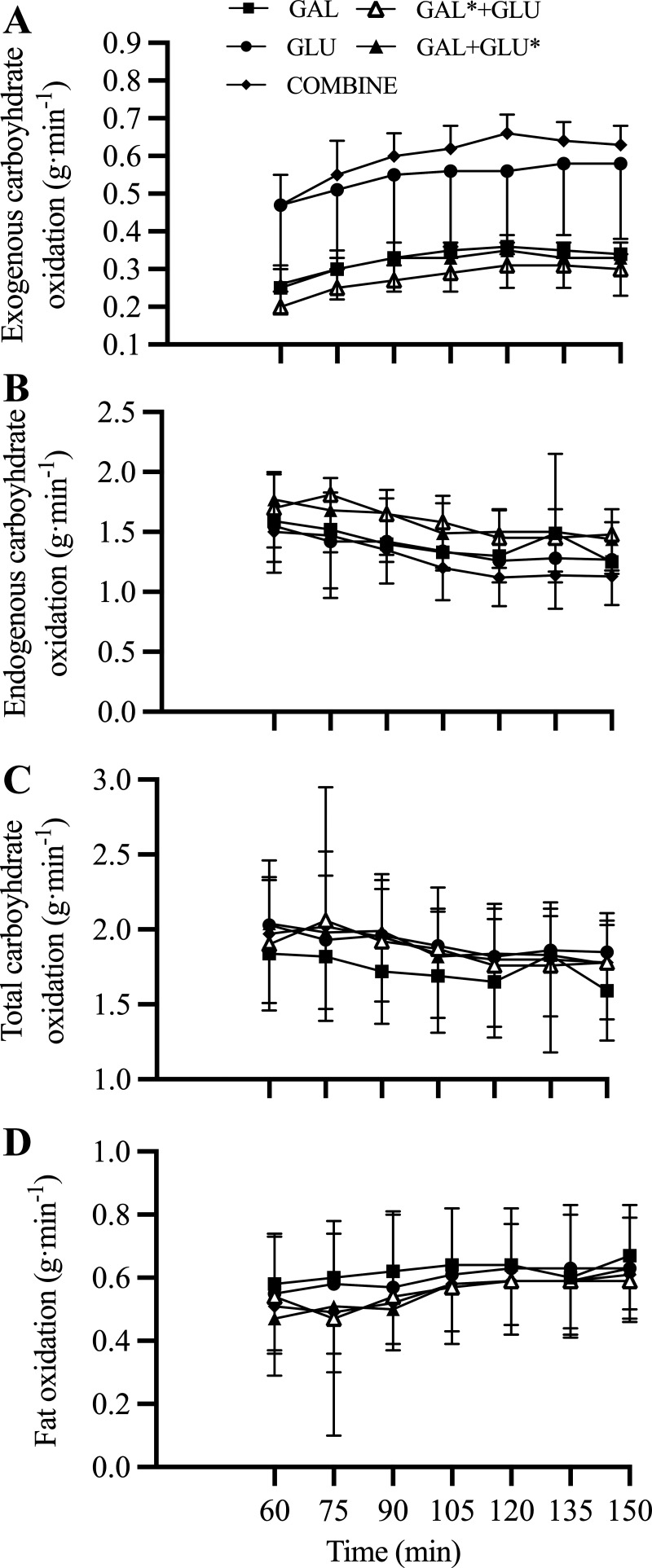
Substrate oxidation during exercise. Exogenous carbohydrate oxidation (*A*), endogenous carbohydrate oxidation (*B*), total carbohydrate oxidation (*C*), and fat oxidation (*D*), during 150 min exercise at 50%W_max_. *n* = 14 (11 male participants, 3 female participants).

Expired breath ^13^CO_2_ enrichment is shown in Fig. 1, with substrate utilization and statistical summary data in Fig. 2 and Table 2, respectively. Resting breath ^13^CO_2_ enrichment was similar in all conditions indicating a similar preexercise endogenous ^13^C status. CHO_exo_ in GAL was lower than in GLU. Coingestion of glucose with galactose (GAL* + GLU) resulted in a minor reduction in CHO_exo_ compared with GAL. CHO_exo_ was not different between GAL* + GLU and GAL + GLU*. CHO_exo_ with ingestion of galactose and glucose (COMBINE) resulted in similar CHO_exo_ rates to GLU. There was no difference in CHO_endo_ rates between GAL and GLU. However, CHO_endo_ was lower in COMBINE than GAL. Fat oxidation was higher in GAL than GAL + GLU*, with no significant difference between GAL and GLU.

**Table 2.** Statistical summary from a repeated-measures linear mixed model analysis of variance of exogenous and endogenous substrate oxidation rates during 150 min of steady-state cycling at 50%W_max_ with the ingestion of differing carbohydrate types

Condition, Comparison	CHO_exo_, g·Min^−1^	CHO_tot_, g·Min^−1^	CHO_endo_, g·Min^−1^	Fat Oxidation, g·Min^−1^
*Least squares mean (95% CL)*				
GAL	0.31 (0.28, 0.35)	1.69 (1.43, 1.94)	1.32 (1.07, 1.58)	0.60 (0.52, 0.68)
GAL* + GLU	0.27 (0.24, 30)	1.70 (1.45, 1.95)	NC	0.55 (0.47, 0.62)
GAL + GLU*	0.30 (0.27, 0.33)	1.83 (1.58, 2.08)	NC	0.52 (0.44, 0.60)
GLU	0.55 (0.52, 0.58)	1.86 (1.61, 2.11)	1.29 (1.04, 1.54)	0.56 (0.48, 0.64)
COMBINE	0.57 (0.49, 0.64)	1.77 (1.49 2.04)	1.18 (0.93, 1.44)	0.53 (0.44, 062)
*Treatment contrasts as least squares mean estimates (adjusted 95% CL); P value*				
GAL* + GLU – GAL	0.05 (−0.09, 0.00); 0.01	0.01 (−0.19, 0.22); 0.89	NC	−0.06 (−0.14, 0.03); 0.17
GAL + GLU* – GAL	−0.01 (−0.06, 0.04); 0.63	0.14 (−0.07, 0.35); 0.08	NC	−0.08 (−0.17, 0.01); 0.02
GLU – GAL	0.24 (0.19, 0.29); <0.0001	0.17 (−0.03, 0.37); 0.02	−0.04 (−0.16, 0.09); 0.50	−0.04 (−0.13, 0.04); 0.31
COMBINE – GAL	0.26 (0.19, 0.32); <0.0001	−0.10 (−0.10, 0.25); 0.28	−0.14 (−0.26, 0.02); 0.02	−0.07 (−0.14, 0.005); 0.02
GAL + GLU* – GAL*+GLU	0.03 (−0.02, 0.08); 0.11	0.13 (−0.09, 0.36); 0.17	NC	−0.03 (−0.13, 0.07); 0.59
GLU – GAL* + GLU	0.28 (0.23, 0.33); <0.0001	0.16 (−0.05, 0.37); 0.04	NC	0.01 (−0.08, 0.10); 0.65
GLU – GAL + GLU*	0.25 (0.19, 0.31); <0.0001	0.03 (−0.21, 0.27); 0.89	NC	0.04 (−0.06, 0.14); 0.42
COMBINE − GLU	0.02 (−0.05, 0.09); 0.63	−0.10 (−0.29, 0.10); 0.23	0.10 (−0.23, 0.02); 0.10	−0.03 (−0.11, 0.05); 0.54

*n* = 14 (11 male participants, 3 female participants). *^13^C-labeling. CHO_endo_, endogenous carbohydrate oxidation; CHO_exo_, exogenous carbohydrate oxidation; CHO_tot_, total carbohydrate oxidation; COMBINE, galactose and glucose coingestion; GAL, galactose; GLU, glucose; NC, not calculated.

### Plasma Metabolites

**Figure 3.**
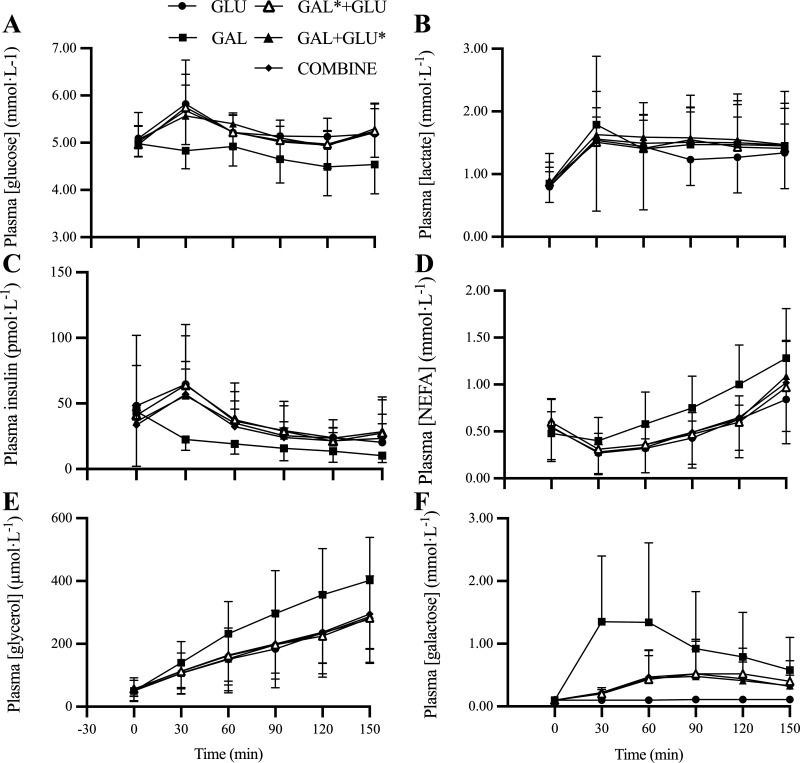
Plasma glucose (*A*), lactate (*B*), insulin (*C*), NEFA (*D*), glycerol (*E*), and galactose (*F*) concentrations during 150 min exercise at 50%W_max_. *n* = 14 (11 male participants, 3 female participants). NEFA, nonesterified fatty acid.

Data for plasma metabolites are *n* = 10–12 due to blood sampling problems in some trials and shown in Fig. 3 with a statistical summary in Table 3. Plasma glucose concentration over the 30–150 min period was significantly lower in GAL than in GAL* + GLU and GAL + GLU*. Plasma lactate was similar in all conditions. Plasma insulin concentrations were lower in GAL than in GLU, GAL* + GLU, and GAL + GLU*. Plasma NEFA concentrations were higher in GAL than GLU, GAL* + GLU, and GAL + GLU*. Similarly, plasma glycerol was higher in GAL than GLU, GAL* + GLU, and GAL + GLU*. Plasma galactose concentrations were higher in GAL than in GLU* + GAL and GAL + GLU*. Galactose concentrations were lower in GLU than in all other conditions.

**Table 3.** Statistical summary from a repeated-measures linear mixed model analysis of variance of plasma metabolite concentrations during the 30–150 min of steady-state cycling at 50%W_max_ with the ingestion of differing carbohydrate types

Condition, Comparison	Glucose, Mmol·L^−1^	Lactate, Mmol·L^−1^	Insulin, Pmol·L^−1^	NEFA, Mmol·L^−1^	Glycerol, µmol·L^−1^	Galactose, Mmol·L^−1^
*Least squares mean (95% CL)*						
GAL	4.8 (4.3, 5.4)	1.5 (1.1, 1.9)	18.8 (6.3, 31.3)	0.9 (0.7, 1.1)	302 (232, 372)	0.9 (0.7,1.2)
GAL* + GLU	5.3 (4.8, 5.8)	1.6 (1.2, 2.0)	35.4 (22.9, 47.9)	0.6 (0.5, 0.8)	230 (161, 299)	0.4 (0.1, 0.6)
GAL + GLU*	5.3 (4.8, 5.8)	1.8 (1.4, 2.2)	39.6 (27.1, 52.1)	0.6 (0.4, 0.7)	209 (141, 278)	0.4 (0.1, 0.6)
GLU	5.4 (4.9, 5.9)	1.4 (1.0, 1.8)	38.2 (25.7, 50.7)	0.6 (0.4, 0.7)	215 (145, 284)	0.1 (−0.2, 0.3)
*Treatment contrasts, least squares means estimates (adjusted 95% CL); P value*						
GAL* + GLU – GAL	0.5 (0.3, 0.8); <0.0001	0.05 (−0.3, 0.4); 0.71	16.0 (6.3, 26.4); 0.0003	−0.2 (−0.4, −0.1); 0.0003	−72 (−120, −23); 0.0007	−0.6 (−0.9, −0.3); <0.0001
GAL + GLU* – GAL	0.5 (0.2, 0.8); <0.0001	0.3 (−0.1, 0.6); 0.26	20.8 (9.0, 32.6); <0.0001	−0.3 (−0.5, −0.2); <0.0001	−92 (−148, −37); 0.0001	−0.6 (−0.9, −0.2); <0.0001
GLU – GAL	0.5 (0.3, 0.9); <0.0001	−0.1 (−0.4, 0.2); 0.45	19.4 (9.7, 29.2); <0.0001	−0.3 (−0.4, −0.2); <0.0001	−87 (−134, −40); <0.0001	−0.9 (−1.2, −0.6); <0.0001
GAL + GLU* – GAL + GLU*	−0.01 (−0.3, 0.3); 0.97	0.2 (−0.2, 0.6); 0.45	4.2 (−8.3, 16.7); 0.64	−0.1 (−0.3, 0.1); 0.24	−20 (−80, 39); 0.64	0.02 (−0.3, 0.4); 0.87
GLU – GAL* + GLU	0.02 (−0.2, 0.3); 0.97	−0.2 (−0.5, 0.2); 0.43	2.8 (−7.6, 13.9); 0.70	−0.1 (−0.2, −0.1); 0.23	−16 (−65, 34); 0.64	−0.3 (−0.6, −0.02); 0.01
GLU – GAL + GLU*	0.03 (−0.3, 0.3); 0.97	−0.4 (−0.8, 0.004); 0.05	−1.4 (−13.9, 11.1); 0.76	−0.01 (−0.2, 0.2); 0.92	5 (−54, 64); 0.82	−0.3 (−0.7, 0.003); 0.02

*n* = 14 (11 male participants, 3 female participants). *^13^C-labeling. COMBINE, galactose and glucose coingestion; GAL, galactose; GLU, glucose; NEFA, nonesterified fatty acid.

### Physiological and Perceptual Characteristics of Exercise Bouts

RPE [mean (SD): GAL, 12 (1); GAL* + GLU, 11 (2); GAL + GLU*, 12 (2); GLU, 12 (1)] and HR [mean (SD): GAL, 139 (14); GAL* + GLU, 137 (13); GAL + GLU*, 140 (15); GLU, 139 (11) b·min^−1^] were comparable between conditions. GI symptoms (i.e., nausea, fullness, and cramping) measured on a 10-point Likert scale were minimal in all conditions (all mean scores for each symptom <2 for all conditions).

## DISCUSSION

In contrast to the study hypothesis, coingestion of glucose with galactose during exercise did not increase exogenous-galactose oxidation rates. Nonetheless, combined galactose-glucose ingestion during exercise resulted in similar CHO_exo_ to the consumption of isoenergetic quantities of glucose alone, suggesting that galactose-glucose blends provide a valid and alternative exogenous-carbohydrate source to glucose.

The hypothesis that coingestion of glucose with galactose would increase the oxidation of exogenous galactose was formulated based on observations made under resting conditions showing glucose and galactose coingestion markedly attenuated the plasma galactose response to galactose feeding via enhanced splanchnic galactose clearance (13, 14). The fate of the enhanced galactose clearance at rest has been attributed to storage of liver glycogen following galactose-to-glucose conversion in the Leloir pathway (14). To our knowledge, the present study demonstrates for the first time that a blunting of galactosemia also occurs during exercise with a ∼60% reduction in plasma-galactose concentrations with combined galactose-glucose ingestion compared with galactose alone. The plasma-galactose concentration response to combined galactose-glucose ingestion during exercise was essentially identical to that observed with lactose ingestion in a previous study using similar experimental conditions (20). Therefore, the data suggest that at rest and during exercise coingestion of glucose with galactose as free monosaccharides or as intact lactose reduces the rise in circulating galactose concentrations seen when galactose alone is ingested. It has been postulated from studies performed at rest that intrahepatic metabolism rather than other factors, such as the increased presence of insulin (systemic or portal) increasing galactose and/or glucose uptake explain the observation (13, 14). Although the precise mechanism, and if differences exist between rest and exercise, is yet to be fully resolved.

Despite glucose and galactose coingestion reducing plasma galactose, this did not translate into enhanced oxidation of ingested galactose during exercise. In fact, coingestion of glucose and galactose resulted in a trivial (0.05 g^.^min^−1^) reduction in exogenous galactose oxidation, which would equate to a difference of ∼6 g over the entire 150 min of exercise. The uncertainty suggests the reduction could range from 0 to 0^.^09 g^.^min^−1^, with the effect size being below the smallest critical value. Although likely, metabolically trivial, the reduction nevertheless, was mostly compensated for by a marginal increase in exogenous-glucose oxidation (0.03 g^.^min^−1^; 95% CL −0.02, 0.08) in the combined drink. Combined galactose-glucose beverages provided carbohydrate ingestion rates at below-saturation quantities for intestinal absorption. Therefore, despite galactose and glucose following identical intestinal transport processes, it seems unlikely that galactose absorption was impeded by the presence of glucose. Rather, the absence of increased oxidation of ingested galactose despite lowering of the plasma galactose response with glucose and galactose coingestion suggests that enhanced splanchnic galactose uptake may have occurred. However, subsequent increased splanchnic galactose-to-glucose conversion could have been directed to liver glycogen synthesis, as postulated to occur at rest (14), rather than contributing to hepatic-glucose output, as we hypothesized would occur in the context of exercise.

It is noteworthy that with respect to CHO_exo_ in GAL, the oxidation of the galactose component in GAL* + GLU and the oxidation of the glucose component in GAL + GLU* all clustered around rates of ∼0.3 g·min^−1^. These data suggest when ingested at rates of 0.4 g·min^−1^ galactose and glucose are oxidized to a similar extent and with similar efficiency (at least when coingested), which is in contrast to the only two other studies that have determined CHO_exo_ from galactose and glucose consumed specifically during exercise. Leijssen et al. (11) observed CHO_exo_ rates of 0.41 (0.03) g·min^−1^ and 0.85 (0.04) g·min^−1^ with galactose or glucose ingestion, respectively when ingesting ∼1.2 g·min^−1^ during exercise. Burelle et al. (9) demonstrated CHO_exo_ rates of glucose and galactose to be 0.53 (0.04) g·min^−1^ and 0.30 (0.05) g·min^−1^, respectively, with ingestion rates of 0.83 g·min^−1^. These previous studies utilized higher ingestion rates of galactose or glucose and the study by Leijssen et al. (11) observed plasma galactose concentrations rose substantially higher than those observed in the present study suggesting considerable limitation in hepatic galactose to glucose conversion at higher galactose intakes. It is possible that in the present study with lower doses of galactose that hepatic galactose metabolism was not so limited and thus produced similar oxidation rates to isoenergetic glucose. Indeed, preexercise feeding of galactose or glucose, which provides time for hepatic-galactose metabolism, resulted in similar oxidation of the two monosaccharides during subsequent exercise (28). The absence of a hepatic limitation in galactose metabolism at the dose used in the present study could also explain why glucose and galactose coingestion did not augment exogenous galactose oxidation. The data are interpreted to mean that at relatively low intakes of galactose or glucose during exercise, their oxidation is similar, but it is acknowledged that a direct comparison of low- or moderate-dose exclusive galactose versus glucose ingestion was not made in the present study. Dose-response studies are now needed to understand at what galactose ingestion rate does the exogenous oxidation rate diverge from that of ingested glucose or other carbohydrate types.

The use of selective isotope tracer labeling of galactose and glucose in the GAL* + GLU and GAL + GLU* trials, respectively, enabled the quantitation of CHO_exo_ from combined galactose-glucose ingestion. Using the approach, it was shown that the mean CHO_exo_ of combined galactose-glucose was comparable with when isoenergetic quantities of glucose alone were ingested (0.57 and 0.55 g·min^−1^, respectively). The exogenous glucose oxidation rates are consistent with a previous study [0.58 (0.05) g·min^−1^] that utilized the same glucose ingestion rate, exercise intensity, and duration (4). The CHO_exo_ of combined galactose and glucose was also similar to CHO_exo_ rates observed with lactose ingestion [0.56 (0.19) g·min^−1^] with identical ingestion rates (20). This supports the notion that digestion of lactose at least in moderate quantities is likely not limiting its oxidation, though a direct comparison between lactose and combined glucose and galactose is needed to confirm this. In the same study, lactose resulted in similar CHO_exo_ rates to sucrose [0.61 (0.10) g·min^−1^]. It would seem, therefore, that sucrose, lactose, glucose, combined galactose-glucose, and combined fructose-glucose can be readily oxidized to a similar extent when ingested at moderate rates (<1.0 g·min^−1^). CHO_exo_ following galactose-glucose ingestion at higher doses (i.e., ≥1.0–1.2 g·min^−1^) remains to be investigated. As intestinal CHO transport is a key limitation to CHO_exo_ and galactose is absorbed in an identical manner to glucose, it is likely that equivalence of CHO_exo_ between the carbohydrates would be limited to moderate ingestion rates (<1.0 g·min^−1^). The similarity in CHO_exo_ with combined galactose-glucose and glucose alone further suggests the small reduction in exogenous-galactose oxidation observed with coingestion of glucose with galactose is unlikely to be physiologically meaningful. Indeed, although not directly comparable, an investigation by Stannard et al. (12) found that although superior to consuming galactose only, performance during a preloaded cycle time trial was similar with combined glucose-galactose ingestion (1:1 ratio) and combined glucose-fructose ingestion (4:1 ratio). Although it remains to be tested, these data are interpreted to mean that the performance benefits from galactose-glucose and glucose-only feeding during exercise could be similar.

In conclusion, glucose and galactose coingestion did not increase the oxidation of ingested galactose during exercise. Combined galactose-glucose ingestion at moderate quantities elicited similar cumulative exogenous carbohydrate oxidation rates as compared with that resulting from ingestion of isoenergetic quantities of glucose.

## GRANTS

This project was supported by a grant from Dairy Management Inc. (to G.A.W. and D.S.R.), which comprises the National Dairy Council, The American Dairy Association, and the U.S. Dairy Export Council.

## DISCLOSURES

O.J.O. receives funding from the Biotechnology and Biological Sciences Research Council (United Kingdom) as part of an iCASE studentship (Midlands Integrative Biosciences Training Partnership), in partnership with Volac International Ltd (United Kingdom). G.A.W. has received research funding and/or has acted as a consultant for GlaxoSmithKline Ltd (United Kingdom), Sugar Nutrition UK, Lucozade Ribena Suntory Ltd (United Kingdom) and Volac International Ltd. D.S.R. has received consultancy research funds from Frucor Suntory Beverages (New Zealand), Zespri Ltd (New Zealand), and Lucozade-Ribena-Suntory Ltd (United Kingdom). 

## AUTHOR CONTRIBUTIONS

S.G.I., D.S.R., and G.A.W. conceived and designed research; O.J.O., S.G.I., B.J.S., T.P., and R.B.S. performed experiments; O.J.O., B.J.S., T.P., D.S.R., and G.A.W. analyzed data; O.J.O., T.P., D.S.R., and G.A.W. interpreted results of experiments; O.J.O. and S.G.I. prepared figures; O.J.O. and G.A.W. drafted manuscript; O.J.O., S.G.I., B.J.S., T.P., R.B.S., D.S.R., and G.A.W. edited and revised manuscript; O.J.O., S.G.I., B.J.S., T.P., R.B.S., D.S.R., and G.A.W. approved final version of manuscript. 
